# Supplementary material for: Quantifying agent impacts on contact sequences in social interactions
Source: Sci Rep. 2022 Mar 3;12:3483. doi: 10.1038/s41598-022-07384-0 (PMC8894368; doi:10.1038/s41598-022-07384-0)
Supplement: Supplementary file 1 — Supplementary Information. [file 41598_2022_7384_MOESM1_ESM.pdf]

Quantifying agent impacts on contact sequences in social interactions

Supplementary Materials

Mark M. Dekker[1,2,*], Tessa F. Blanken[3], Fabian Dablander[3], Jiamin Ou[1,4], Denny Borsboom[3], and Debabrata Panja[1,2]

[1]Department of Information and Computing Sciences, Utrecht University, Princetonplein 5, 3584 CC Utrecht, The Netherlands
[2]Centre for Complex Systems Studies, Utrecht University, Minnaertgebouw, Leuvenlaan 4, 3584 CE Utrecht, The Netherlands
[3]Department of Psychological Methods, University of Amsterdam, Nieuwe Achtergracht 129-B, 1018 VZ Amsterdam, The Netherlands
[4]Department of Sociology, Utrecht University, Padualaan 14, 3584 CH Utrecht, the Netherlands
[*]m.m.dekker@uu.nl

December 15, 2021

A Supplementary Plots

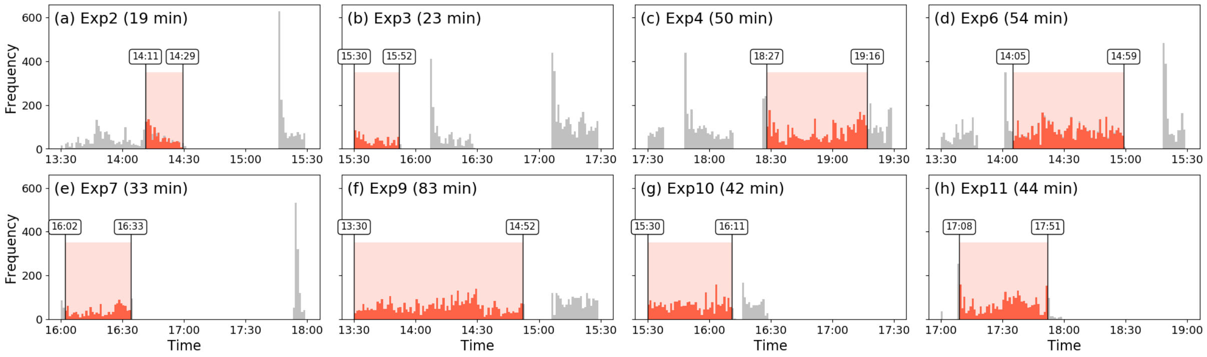

Figure A.1: Histograms of the time stamps of events across the eight experiments. In red, the filtered windows used in this paper are highlighted.

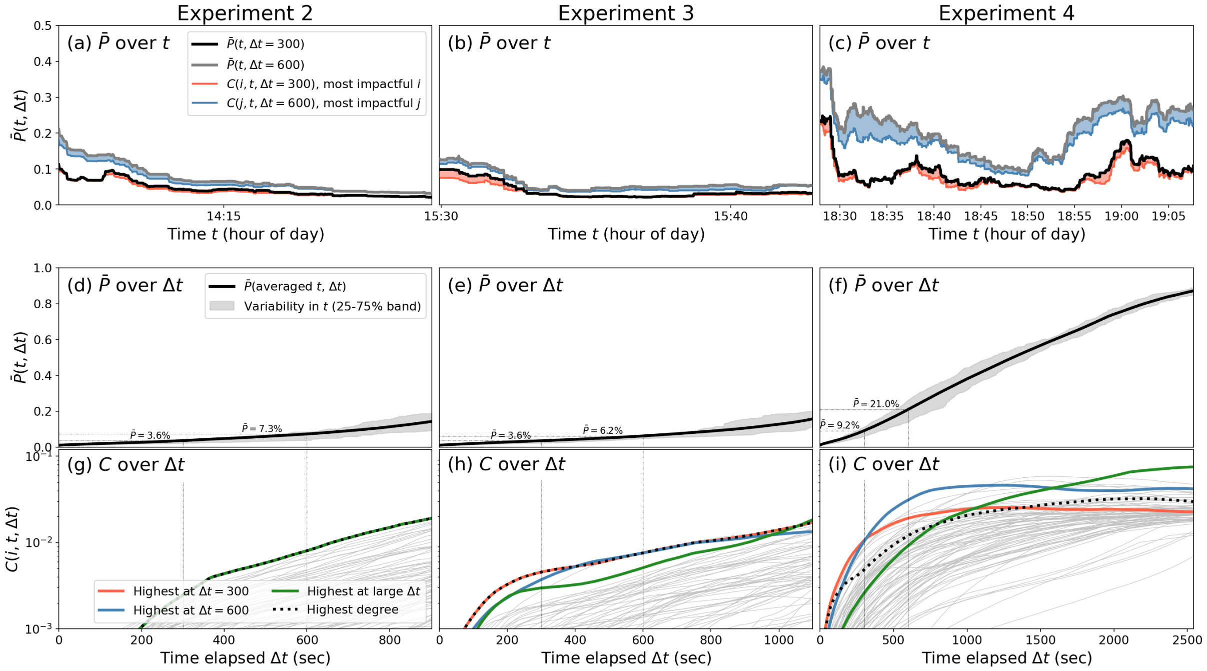

Figure A.2: Same as Fig. 3 in the main text, but for experiments 2, 3 and 4.
